# Mid-term results after DAIR for patients with acute periprosthetic joint infections of the hip or knee

**DOI:** 10.1186/s13018-025-06117-z

**Published:** 2025-07-18

**Authors:** Alberto Alfieri Zellner, Niclas Watzlawik, Jonas Roos, Gunnar Thorben Rembert Hischebeth, Christian Prangenberg, Alexander Franz, Frank Sebastian Fröschen

**Affiliations:** 1 Department. of Orthopedics and Trauma Surgery, University Hospital of Bonn, 53127 Bonn, Germany; 2 Institute of Medical Microbiology, Immunology and Parasitology, University Clinic of Bonn, Bonn, Germany; 3 Department of Trauma and Orthopedic Surgery, BG Klinik Ludwigshafen, 67071 Ludwigshafen, Germany

**Keywords:** DAIR, PJI, Acute periprosthetic infection, Revision-free survival, Arthroplasty

## Abstract

**Introduction:**

Periprosthetic joint infections (PJIs) are severe complications following total joint arthroplasty, with significant implications for implant longevity and patient quality of life. The debridement, antibiotics, irrigation, and implant retention (DAIR) procedure is a key strategy for managing acute PJIs while preserving the prosthesis. However, its success is highly variable, influenced by factors such as pathogen virulence and patient-specific risks. We set out to evaluate revision-free implant survival and potential risk factors influencing outcome at our institution.

**Materials and methods:**

This retrospective, single-center study analyzed a total of 110 patients (60 hip and 50 knee) treated for acute periprosthetic joint infections (PJI) with DAIR between 2017 and 2022. Exchange of mobile parts was undertaken in all cases. Postoperative management followed a standardized protocol, consisting of two weeks of intravenous antibiotics followed by four weeks of oral antibiotics. Clinical and radiological follow-ups were conducted at predefined intervals, assessing implant stability and signs of reinfection. Comprehensive patient data, including demographics, infection markers, microbiology, implant type, and prior surgical history, were collected and analyzed statistically.

**Results:**

Overall, 23.6% (*n* = 26) of patients were lost to follow-up. Of the remaining 84 patients, we were able to detect 31 cases of tier 1 success according to Fillingham outcome criteria, which represents 36.9%. The patients in whom DAIR failed, tended to be older, have more comorbidities and showed a higher total cell count in preoperative joint aspiration. Furthermore, prior revision arthroplasty was associated with a significantly higher failure rate in the knee group.

**Conclusion:**

A preoperative assessment of the likelihood of DAIR success should be undertaken for each patient. For this assessment, our data indicates to look at patient specific factors such as age, ASA score, revision implant, and preoperative cell count. These aspects may enhance risk evaluation and support the selection of an alternative treatment strategy when appropriate.

## Introduction

Periprosthetic joint infections (PJIs) are among the most serious complications following total joint arthroplasty, with a reported incidence of 1–2% in primary joint replacements and up to 10% for revision arthroplasties [1–7]. PJIs pose a significant challenge due to their potential to compromise implant longevity, patient morbidity and reduce mobility, and negatively impact quality of life [8–12]. The rationale for implant retention lies in the preservation of joint function and avoiding the morbidity associated with immobilization or joint resection. The management of PJIs is complex, involving a delicate balance between eradicating infection and preserving joint function, often requiring a combination of surgical and medical interventions.

The debridement, antibiotics, irrigation, and implant retention (DAIR) procedure is characterized by surgical debridement of the surrounding infected tissue, exchange of the mobile parts of the inlying implants, intraoperative irrigation, and postoperative targeted antibiotic therapy, with the aim of retaining the prosthetic components.

One of the critical challenges in managing PJIs is the formation of bacterial biofilms on the prosthetic surface, which protect the causative pathogens from antibiotics and the host`s immune defenses [13]. The DAIR procedure aims to prevent the formation of a mature biofilm by early surgical intervention and the use of biofilm-active antibiotics after mechanical debridement. It is believed that exchanging the mobile parts of the prosthesis during surgery can improve the outcome when treating PJIs [14, 15]. However, once biofilm formation and maturation have occurred, the likelihood of successful infection control decreases significantly. Therefore, the DAIR procedure is a treatment option for early or acute late-onset PJIs, where biofilm formation on the surface of the implant is thought to be in its initial stages and more amenable to eradication. In vitro studies have shown that biofilm formation begins within hours after infection, which further complicates treatment [16]. Therefore, targeted antibiotic therapy should be combined with antibiofilm therapy (usually using Rifampicin for Gram-positive bacteria and usually done with flouroquinolones for Gram-negative bacteria) early on, while the biofilm is amenable to eradication [17]. It is strongly recommended that, when treating PJIs, an interdisciplinary team with microbiologists and/or infectious disease experts should work on treating this challenging disease. Resistance to Rifampicin, for instance, can significantly impact the outcome when treating PJIs [18, 19]. However, despite its relative success in early infections, DAIR has shown mixed outcomes, with reported success rates ranging from 50 to 90% [20–22]. This variability is largely influenced by factors such as the timing of intervention, pathogen virulence, host immune response, and adherence to a strict postoperative antibiotic regimen. In general the evaluation of risk factors is essential to improve treatment, especially in vulnerable or elderly patients regardless of the performed procedure [23–25]. Diabetes and polymicrobial infections have been identified as independent risk factors for DAIR failure [26, 27].

The aim of the study was to provide mid-term data on revision-free implant survival of infected total hip arthroplasty (THA) and total knee arthroplasty (TKA) after DAIR, and to evaluate the impact of patient-specific risk factors on revision-free implant survival.

By understanding these variables, we aim to provide insights that may help improve patient selection, refine surgical techniques, and optimize antibiotic strategies, ultimately contributing to better management protocols for the treatment of acute PJIs with DAIR.

## Materials and methods

We conducted a retrospective study that included patients who were treated in our department from 2017 to 2022 for an acute periprosthetic infection requiring DAIR of the hip or knee. We included 110 patients (60 (54,5%) THA and 50 (45,5%) TKA). We differentiated between acute early onset infection (infection within 6 weeks postoperatively with symptoms lasting less than three weeks), acute late onset infection (infection more than 6 weeks postoperatively with symptoms lasting less than three weeks) and acute hematogenous infection (positive blood culture samples with symptoms lasting less than three weeks) following an adapted version of the Tsukayama and Izakovicova classifications [28, 29]. Exclusion criteria were presence of a chronic infection defined as: presence of a sinus tract communicating with the prosthesis, symptom duration of > 3 weeks, or loosening of the inlying prosthesis.

Intraoperative samples (tissue biopsy, synovial fluid and sonication) were obtained from all included patients, and subsequently analyzed in the microbiological department and pathology department.

For diagnosing a PJI the following criteria defined by Parvizi et al. (2018) were used:

- isolation of the same microorganism from two or more cultures/tissue biopsies obtained from the infected joint.
- isolation of one microorganism in the intraoperative cultures with additional evidence of an infection of the inlying implant (positive histology, presence of purulence, elevated serum erythrocyte sedimentation rate, elevated C-reactive protein, and elevated synovial white blood cell count.

In summary, only patients with an acute periprosthetic joint infection and need for DAIR to retain the prothesis were included [30].

All patients were treated postoperatively following a standardized algorithm which, following DAIR, included 2 weeks of targeted intravenous (i.v.) antibiotic therapy followed by 4 weeks of oral antibiotics. Furthermore, we performed clinical and radiological follow-ups after 6 weeks, 6 months and 12 months and annual follow-ups after that. In the radiological follow-ups special attention was paid to new or progressive radiolucent lines greater than 2 mm as a sign of loosening. In cases where a recurrent infection was suspected, we carried out blood analysis for inflammatory markers and performed joint aspirations to analyze the fluid for total cell count and microbiological growth (culture and polymerase chain reaction).

For histology, the samples were identified following the histopathological consensus classification of the periprosthetic interface membrane by Krenn and Morawietz, which differentiates between (particle type (type I), infectious type (type II), combined type (type III) and indifferent type (type IV)) [31].

To provide a more detailed characterization of the included patients, we collected demographic and clinical data, including weight, the site of arthroplasty (hip or knee), preoperative synovial cell count, microbiology of joint aspiration, preoperative white blood cell count and C-reactive protein levels, comorbidities, BMI, the specific procedure performed, and the number of previous surgeries) as well as the type of inlying implant. A polymicrobial PJI was defined as the isolation of more than one microorganism from intraoperative tissue biopsies, sonication fluid, or synovial fluid. We differentiated between primary implants and revision implants. Revision implants were defined as modular implants (used in revision arthroplasty or in the presence of severe bone defects) as well as tumor protheses (bone-replacing implants e.g. proximal/distal femur replacement after tumor resection).

Data were compiled using Microsoft Excel 2024 (Microsoft Corporation, Richmond, VA, USA). Statistical analyses were conducted with SPSS Statistics version 28 for Windows (SPSS, Inc., an IBM company, Chicago, IL, USA). Descriptive statistics, including arithmetic means, standard deviations, and ranges were calculated. Results are presented as means ± standard deviation (SD), unless otherwise stated.

The data was analyzed using the Chi-squared test with Cramérs V to test for associations between nominal variables. A Kaplan-Meier analysis was performed to evaluate revision-free implant survival. Patients lost to follow-up were censored at last contact for Kaplan-Meier analysis. We aimed to examine the association between revision-free implant survival and patient-associated risk factors (comorbidities). The measured continuous variables that represent risk factors included BMI, preoperative C-reactive protein levels and white blood cell count, preoperative joint aspiration cell count and microbiology from joint aspiration, number of previous surgeries and the duration of symptoms. As age was found to be normally distributed according to a Shapiro-Wilk-test, the p-value was calculated using an unpaired, two tailed t-test. The p-value for the BMI and number of prior operations before DAIR was calculated using a Mann-Whitney-U test, as these variables were found to be not normally distributed in the Shapiro-Wilk test. The number of patients was tested for binomial distribution. The ASA-Score was analyzed for significant difference using a Chi² test, as was the presence of different comorbidities.

## Results

We were able to include 110 patients subdivided into 60 PJIs of the hip and 50 PJIs of the knee. All patients were treated with DAIR following a standardized postoperative protocol. Further demographic data is provided in Table 1 below.

**Table 1:** Demographic data of the hip and knee PJI groups

Demographic data	Hip	Knee	Total
Total Number of Patients	60 (54.5%)	50 (45.5%)	110
male female	25 (50%) 35 (58.3%)	25 (50%) 25 (41.7%)	50 60
Age, mean ± SD, (Range)	70.45 ± 11.23 (48–94)	70.38 ± 10.35 (42–97)	70.43 ± 10.91 (42–97)
BMI [kg/m²], mean ± SD, (Range)	30.66 ± 8.03 (18.4–49.9)	31.55 ± 7.01 (21.2–45.9)	31.01 ± 7.58 (18.4–49.9)
Primary implant Revision implant Tumor/Megaprosthesis	21 (52.5%) 39 (56.5%) 0 (0%)	19 (47.5%) 30 (43.5%) 1 (100%)	40 69 1
Nr. of Operations before DAIR, mean ± SD, (Range)	2.49 ± 2.66 (0–10)	2.77 ± 3.20 (0–11)	2.57 ± 2.87 (0–11)

We also analyzed the comorbidities of the two subgroups. Patients with COPD (*p =* 0.002) and cortisone therapy (*p* = 0.003) were significantly more prevalent in the knee group.

Most other comorbidities were more prevalent in the knee PJI group and as demonstrated in Table 2.

**Table 2:** Comorbidities of the hip and knee PJI groups; ASA: American society of anesthesiologists; COPD: chronic obstructive lung disease; *two of these patients required Hemodialysis

Comorbidities of the subgroups	Hip	Knee	*p* value
ASA-Score, Median	2	3	0.489
1 (%)	1 (1.7%)	1 (2%)	
2 (%)	30 (50%)	18 (36%)	
3 (%)	28 (46.6%)	29 (58%)	
4 (%)	1 (1.7%)	2 (4%)	
Heart disease	39 (65%)	25 (50%)	0.112
Diabetes mellitus	20 (33.3%)	15 (30%)	0.709
COPD	3 (5%)	13 (26%)	**0.002**
Cortisone therapy	4 (6.7%)	14 (28%)	**0.003**
Renal insufficiency	10 (16.7%)	16 (32%)*	0.089
Rheumatoid arthritis	4 (6.7%)	2 (4%)	0.54
Liver disease	6 (10%)	5 (10%)	1
Neurological diseases	20 (33.3%)	12 (24%)	0.283
Cognitive impairment	3 (5%)	2 (4%)	0.802

We found that the most frequent type of infection following the Tsukayama classification were acute early onset infections in the hip group, whereas the most common type of infection in the knee group was the acute, late onset infection. In total, we were able to find significant differences in the type of infection in the two groups which are demonstrated in Table 3.

**Table 3:** Type of infection following the Tsukayama classification in hip and knee

Tsukayama Classification	Hip	Knee	*p* value
1: positive Microbiology in aseptic revision	3 (5%)	8 (16%)	
2: acute, early onset infection	38 (63.3%)	11 (22%)	**< 0**,**001**
3: acute, late onset infection	19 (31.7%)	24 (48%)	
4: acute hematogenous infection	0 (0%)	7 (14%)	

During the preoperative preparation of the patient, blood work was performed, and joint fluid was aspirated from the symptomatic joint. Table 4 shows the preoperative values for these preoperative exams.

**Table 4:** Preoperative laboratory parameters including CRP and synovial cell count; CRP: C-reactive protein

	Hip	Knee	*p* value
Preoperative CRP [mg/dl], mean ± SD	102.58 ± 87.16	91.91 ± 95.63	0.241
synovial cell count, mean ± SD			
Total cell count/µl, mean ± SD	93,910 ± 117,645	85,559 ± 125,304	0.832
Leucocytes/µl, mean ± SD	93,221 ± 116,352	83,367 ± 120,287	0.795
Granulocytes/µl, mean ± SD	67,575 ± 71,269	60,795 ± 85,526	0.430
Proportion of Granulocytes to Leukocytes in %, mean ± SD	79.33 ± 21.42	70.44 ± 30.01	0.191

During surgery, tissue samples were collected for histopathological analysis following the Krenn and Morawietz classification for periprosthetic membranes [31]. The results of these findings are listed in Table 5. The most common type of membrane was found to be the type II membrane in the hip and knee (51% and 33.3% respectively), which is in line with type II being the infectious type. In the hip, the combined (19.6%) and the indifferent type (19.6%) were the second most common, followed by the particle type (9.8%). In the knee, the differences were even more subtle among the remaining findings, as the indifferent type accounted for 26.7%, and both the particle type and combined type accounted for 20% each.

**Table 5:** Histology according to the Krenn & Morawietz classification for periprosthetic membranes [26], % of the prosthesis

	Hip	Knee	*p* value
Typ 1, Particle type	5 (9.8%)	9 (20%)	
Typ 2, Infectious type	26 (51%)	15 (33.3%)	0.265
Typ 3, Combined type (1 & 2)	10 (19.6%)	9 (20%)	
Typ 4, Indifferent type	10 (19.6%)	12 (26.7%)	

The duration of symptoms prior to admission/surgery was found to be not naturally distributed in Shapiro-Wilk-testing (*p* < 0.001). Therefore, a Mann-Whitney-U test was conducted, and no significant association between duration of symptoms and treatment failure was found (*p* = 0.841).

We found a significant association between ASA-score and DAIR outcome, with a significantly higher failure rate in patients with a higher ASA-score (*p* = 0.015). Meanwhile, we were not able to detect a significant association between BMI and DAIR outcome (*p =* 0.708). Preoperative CRP and synovial cell count were found to be not normally distributed. In the entire cohort, no significant difference between successful and failed DAIR cases was observed (*p* = 0.510 and 0.547 respectively). However, this analysis was based on the subgroup with complete follow-up (*n* = 84), as outcome could not be defined for the 26 patients lost to follow-up. Furthermore Hosmer-Lemeshow testing found that each of these variables them individually, as well as when combined, to be insufficient predictors of treatment success in linear regression modeling.

**Fig. 1:**
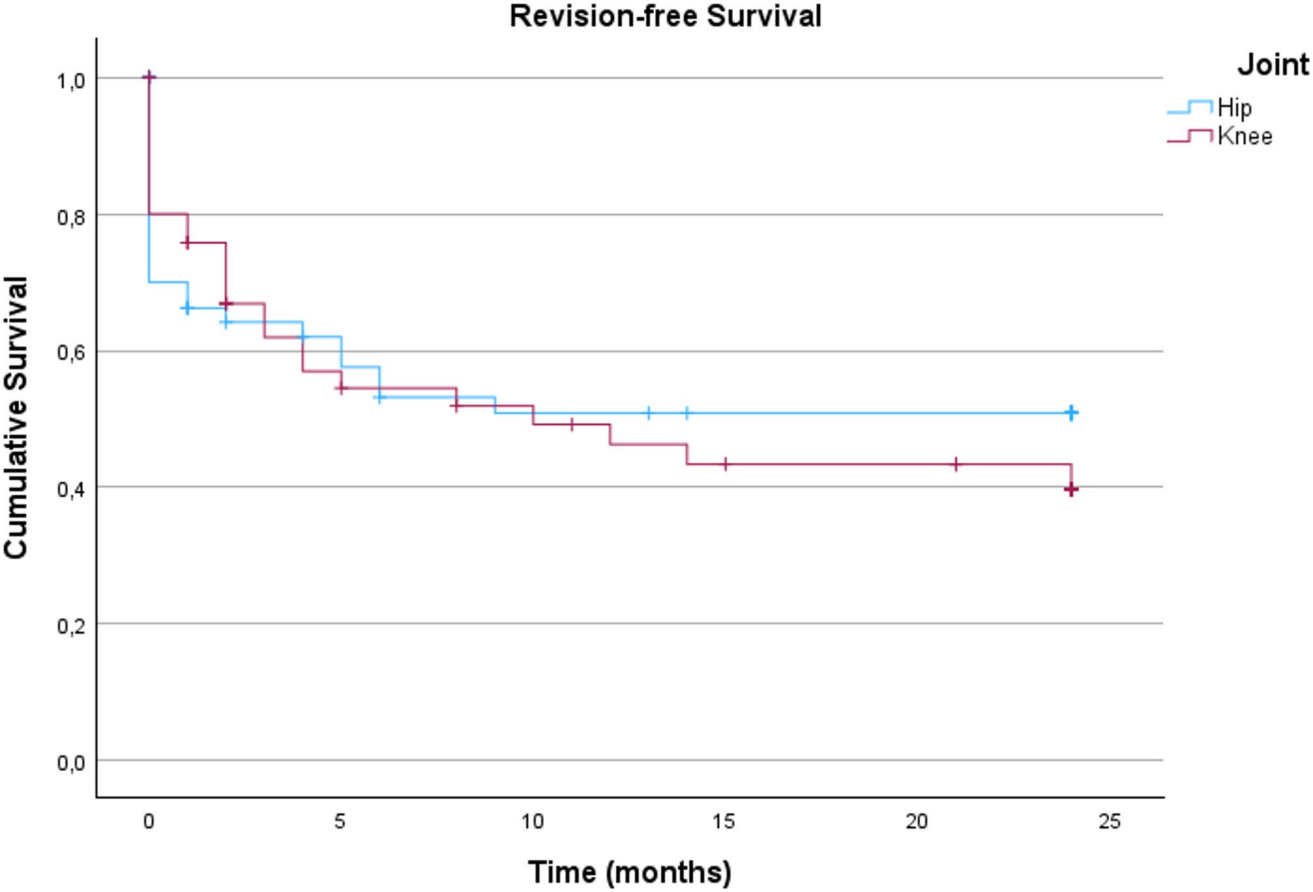
Kaplan-Meier-Survival analysis with the endpoint revision-free implant survival for patients following debridement, antibiotics and implant retention of the total hip arthroplasty and total knee arthroplasty groups (n_total_=110; n_hip_=60, n_knee_=50)

In addition, the revision-free survival of the inlying prosthesis following the DAIR procedure was evaluated. The Kaplan-Meier survival analysis, as shown in Fig. 1, showed no significant differences in the revision-free survival (*p* = 0.674) between the hip and knee subgroups (Table 6).

**Table 6:** Number of patients with revision-free implant survival after 6, 12 and 24 months in the hip and knee subgroups

	Hip (*n* = 60)	Knee (*n* = 50)
6 Months	23 (46.9%)	21 (50%)
12 Months	22 (44.9%)	16 (40%)
24 Month	20 (42.6%)	11 (29.7%)

**Fig. 2:**
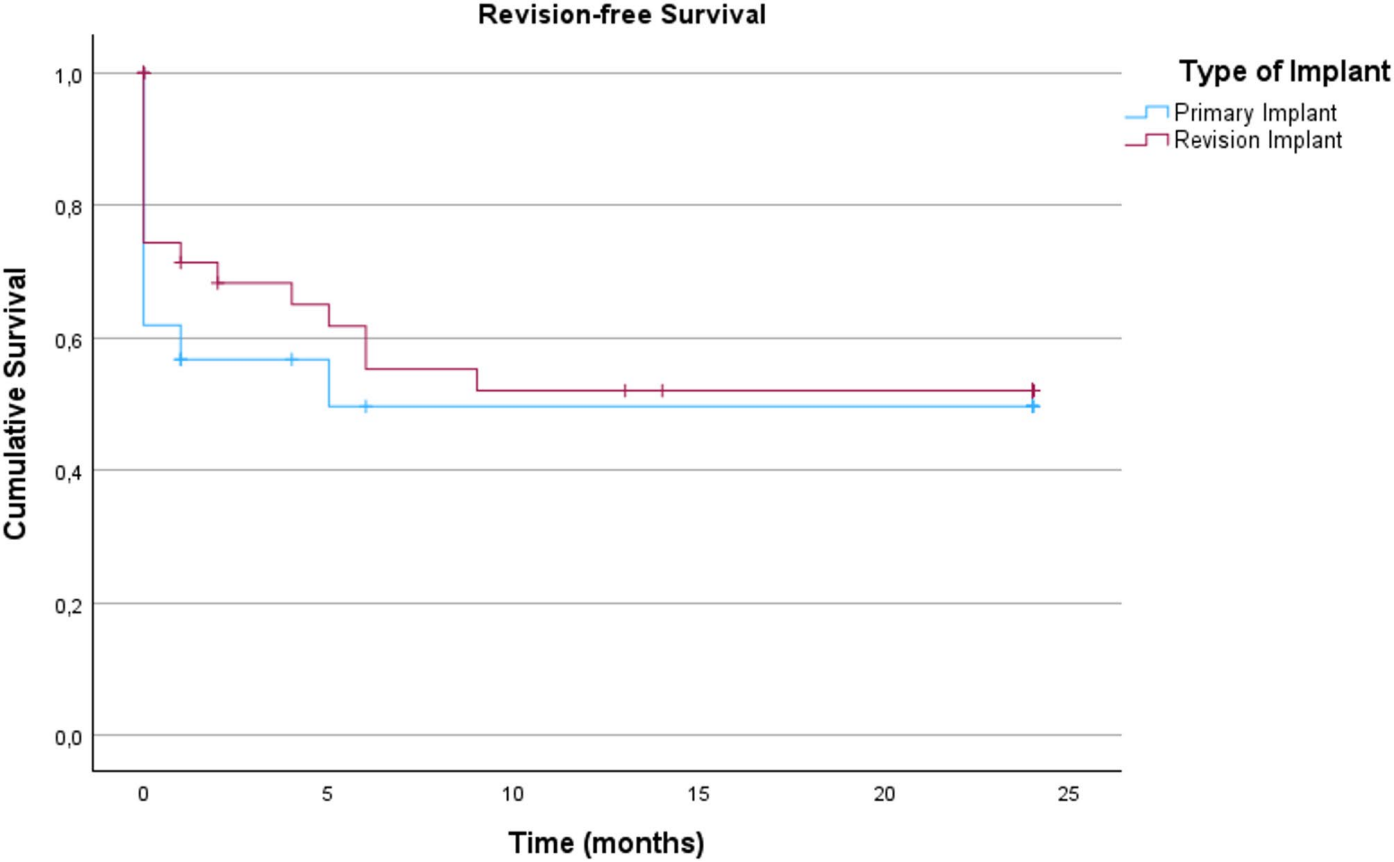
Revision-free implant survival in patients with total hip arthroplasty. (n_total_=60; n_primay_=21, n_revision_=39)

**Fig. 3:**
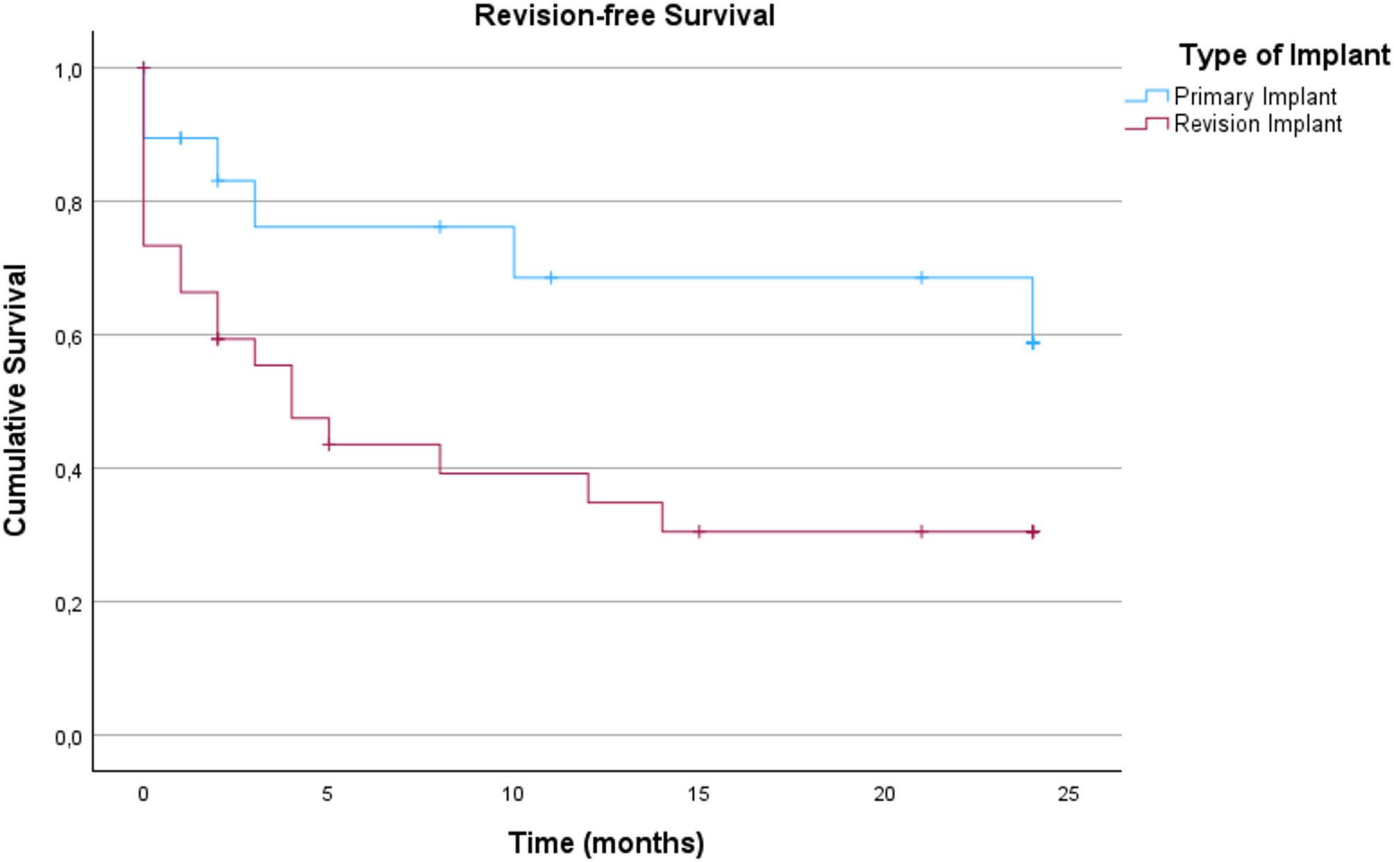
Revision-free implant survival in patients with total knee arthroplasty (n_total_=49; n_TKAprimary_=19, n_TKArevision_=30)

**Fig. 4:**
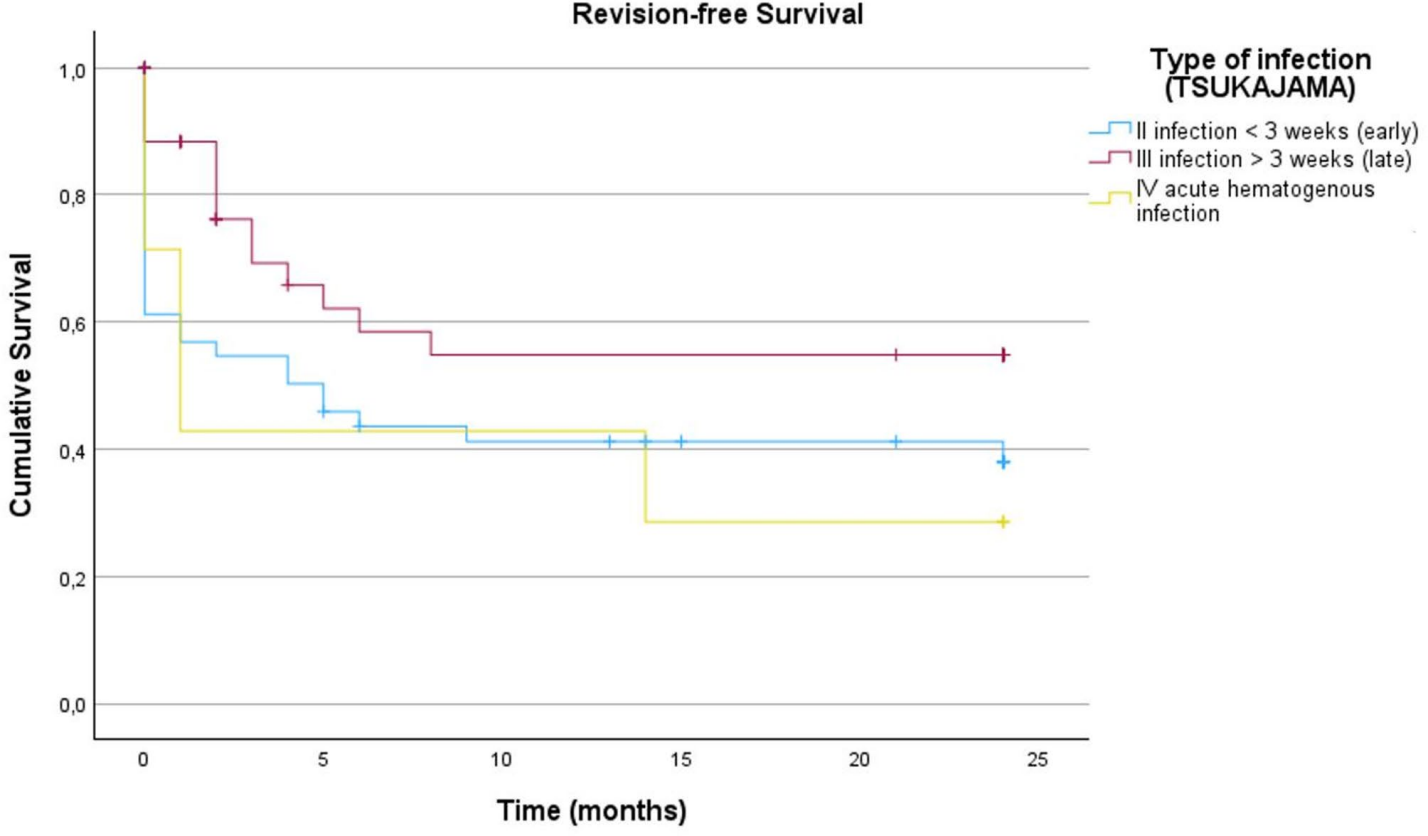
Revision-free implant for subgroup analysis according to type of infection following the Tsukayama classification in hip and knee

For subgroup analysis of the inlying implants see Fig. 2. Here, in the hip PJI group, again, no significant differences were detected (*p =* 0.613) for primary vs. revision implants. In the knee PJI group however, a revision implant undergoing DAIR had a significantly worse outcome in comparison to a primary implant (*p =* 0.039). The differences are demonstrated in Figs. 2 and 3 (the one case of tumor prosthesis was not accounted for in the knee group). For subgroup analysis according to type of infection following the Tsukayama classification in hip and knee (Fig. 4), we could not detect a significant difference in revision-free implant survival (*p* = 0.92).

The subgroup analysis of the preoperative laboratory work-up and risk factors for the knee group are demonstrated in Table 7.

**Table 7:** Subgroup analysis of demographic and laboratory parameters of patients with PJI of inlying (R)TKA (n_total_=49 consisting of n_TKAprimary_=19, n_TKArevision_=30)

Measurement	Primary	Revision	*p*-value
Age at DAIR (mean ± SD)	64.84 (± 8.50)	73.83 (± 10.19)	**0.001**
Preoperative CRP (mg/dl)	72.84 (± 78.55)	106.49 (± 104.76)	0.218
Preoperative Synovial Cellcount	29,110 (± 35094)	112,556 (± 143655)	**0.018**
% of Granulocytes from Leucocytes	68%	71.6%	0.348
Body-Mass-Index	31.76 (± 6.07)	31.65 (± 7.82)	0.751

The reasons for the inlying revision arthroplasty were similarly distributed in the hip and knee group and are demonstrated in the following Table 8.

**Table 8:** Reasons for inlying revision implant in the hip (*n* = 39) and knee (*n* = 30) groups

Reason for revision implant	Hip (*n* = 39)	Knee (*n* = 30)
Aseptic revision in the past	3 (7.7%)	3 (10%)
Fracture	13 (33.3%)	10 (33.3%)
Septic single, two- or multi-stage revision in past	23 (59%)	17 (56.7%)

### Outcome defined by fillingham et al. (2019) after 24-month follow-up

At the follow up of > 24 months, *n* = 26 patients (23.6%) were lost to follow-up. Of the remaining *n* = 84 patients, we detected 31 cases of tier 1 (success) representing 36.9% of the patients available for follow-up. In the subsequently shown Table 9 the different outcome tiers are listed. The most common cause for treatment failure and therefore need for revision of the inlying implant after DAIR surgery was tier 3D (septic revision < 1 year after initial DAIR), followed by a multistage revision needing an amputation, resection arthroplasty or arthrodesis during its treatment course (tier 3E). We observed seven aseptic revisions in the first two years following DAIR surgery. Furthermore, we detected PJI-related death in *n* = 4 patients, representing 4.8% of the follow up group in the first two years following DAIR. Two of these patients suffered from an acute early onset infection and further two patients had an acute hematogenous infection. In total, three patients died on the intensive care unit due to multiorgan failure and sepsis. One patient was transferred to a palliative ward with sepsis following hip PJI, as the patient did not consent to treatment on intensive care unit. The patient died with best supportive care.

**Table 9:** Outcome defined by fillingham et al. (2019)

Outcome Tier	Hip	Knee	Cumulative Percentage
Lost to follow-up	13	13	23.6
Tier 1: Success	20	11	51.8
Tier 3B: septic revision > 1 year after DAIR	0	1	52.7
Tier 3 C: aseptic revision < 1 year after DAIR	4	3	59.1
Tier 3D: septic revision < 1 year after DAIR	9	12	78.2
Tier 3E: amputation, resection arthroplasty (Girdlestone) or arthrodesis	11	7	94.5
Tier 4 A: Death < 1 year after DAIR	1	2	97.3
Tier 4B: Death > 1 year after DAIR	0	1	98.2
Tier 5: chronic sinus tract	2	0	100.0
Total	110		

## Discussion

### Cohort size

Our cohort of 110 patients is among the largest for a 5-year period in comparable studies analyzing outcomes after DAIR for single center studies [32–35]. These studies included patients over a period of 13, 5, 9 and 11 years respectively. Most comparable retrospective studies included fewer patients per year. Our study’s shorter five-year timeframe offers certain advantages, including a more consistent dataset with an almost identical interdisciplinary team. Nonetheless, it remains inferior to studies with a prospective study protocol such as Karlsen et al. (2025) [36], but still demonstrates similar findings: in patients with inlying revision implants and acute PJI, the success of DAIR is limited. Collecting large case numbers over extended periods introduces variability in treatment protocols due to evolving first-line antibiotic therapies, staff turnover, and differences in expertise across different centers.

### Risk factors for recurrence of PJI

In line with Ekhtiara et al. (2022), our data show that advanced patient’s age at the time of revision and a higher ASA score are associated with shorter revision-free implant survival following DAIR [37]. This correlation has also been described by Fink et al. (2017) and Buller et al. (2012) [38, 39]. Our Cohort, which was older and had higher ASA scores than those in comparable studies, thus confirms these findings. These non-modifiable factors contribute to the high failure rates observed in our study, with revision-free survival at 24 months of only 42.6% for hip group and 29.7% for the knee group.

As expected, and in line with current evidence, out results confirm that the synovial cell count is an established diagnostic parameter to support the indication for surgery but does not reliably predict DAIR outcome [40]. The trend described by Ashkenazi et al. (2024) toward higher failure rates in patients with elevated polymorphonuclear cell counts highlights that further prospective research may be warranted to refine prognostic markers, but current evidence does not support routine use of cell count for outcome prediction [40].

In our cohort, patients with revision total knee arthroplasties undergoing DAIR were significantly older and had higher synovial cell counts at diagnosis, confirming the findings of Ekhtiari et al. (2023) that higher age is associated with poorer outcomes, while the elevated cell count reflects its diagnostic rather than prognostic value [37].With age being a non-modifiable risk factor for the recurrence of PJI, and considering that revision implants and megaprostheses (tumor implants) are associated with an increased risk of infection, these findings are consistent with the current literature [40]. The prospective multicenter study by Karlsen et al. (2025), however, demonstrated significantly poorer DAIR outcomes in patients with revision arthroplasty (success rate 65%) compared to primary arthroplasty (success rate 89%) [36]. In contrast, a retrospective study by Nurmohamed et al. (2021) with a comparatively small sample size (*n* = 67) found no significant differences between patients undergoing DAIR following primary or revision total hip or knee arthroplasty, reporting success rates ranging from 56 to 69%. It should be noted, however, that the endpoint was infection control (defined as absence of clinical, laboratory and radiological signs for a PJI) one year postoperatively. In addition, they only included *n* = 16 patients with inlying revision arthroplasties, which has to be considered during outcome comparison [41]. In our analysis a total of *n* = 69 (*n* = 39 hip and *n* = 30 knee) revision arthroplasties were included, which may have affected the outcome of the group. In contrast to the knee subgroup, the hip subgroup did not show significant differences in revision-free implant survival between patients with inlying primary/standard implant and those with inlying revision implant. As hip revision implants may differ significantly in size and include modular components, our data remain unclear regarding outcome evaluation and in comparison with other cohorts. As recommended by Grammatopoulos et al. (2017) we performed the exchange of modular components in 100% of the cases treated with DAIR in our cohort, which eliminates differences that may stem from this aspect [42, 43].

When it comes to prognostic factors, the timing of intervention may play a crucial role. A short duration between onset of symptoms and time to surgery (DAIR) is typically regarded as the most favorable factors for successful infection eradication [44, 45]. In our cohort, no association was found between the duration of symptoms prior to DAIR and the outcome. This may be due to strict indications for surgery which ruled out DAIR in any patients with symptoms lasting more than 4 weeks. Fink et al. (2017) reported an increased success rate of DAIR when surgery was performed no longer than two days after onset of symptoms [38]. These results are consistent with the data from Brandt et al. (1997), who recommended that DAIR surgery should take place two days after the onset of symptoms at the latest [46]. Nevertheless, DAIR surgery might be performed up to a symptom duration of 12 weeks [38].

In addition, patients undergoing DAIR in our cohort had a history of previous surgeries (mean number of surgeries: hip group: 2.49; knee group: 2.77), which is a well-known risk factor for persistent infection after DAIR and in line with current studies by Buller et al. (2012) and Fink et al. (2017) [38, 39].

### Tsukayama classification

Studies have consistently demonstrated that DAIR is most effective in cases of acute early-onset periprosthetic joint infections. For example, Rahardja et al. (2023) reported that DAIR success rates are highest when performed within one month of the primary implant procedure, but they decline significantly to 35% when conducted 24 months postoperatively [47]. Similarly, Wouthuyzen-Bakker et al. (2019) observed a decrease in DAIR success from 74% in early infections to 58% in late infections [48]. In our cohort, 53 patients (48.2%) presented with acute late-onset infections according to the Tsukayama classification. This high proportion of acute late-onset infections, together with the advanced age, higher comorbidity burden, and the frequent presence of revision implants, likely contributes to the comparatively lower DAIR success rates observed in our study compared to the existing literature. Within our cohort, the distinction between early postoperative, late acute, and hematogenous infections did not significantly influence the success of DAIR. For this topic literature shows mixed results regarding the impact of infection type. Some authors report a higher failure rate for hematogenous infections due to delayed diagnosis and more mature biofilm formation, whereas others found no significant difference when strict surgical and antibiotic protocols were applied [43–45]. Our findings align with the latter, supporting the idea that standardized treatment pathways and prompt intervention may mitigate the negative impact of infection type on DAIR outcomes [49].

### Krenn and Morawietz

The histopathological analysis of the periprosthetic membranes in our cohort revealed that, while the majority of cases were classified as type II (infectious type) according to Krenn and Morawietz [31], a notable proportion showed type III (combined) or type IV (indifferent) membranes. This finding might initially appear contradictory given that all cases were confirmed PJIs. A possible explanation for the heterogeneity of infection patterns within the periprosthetic membrane could be, that if biopsies are also taken from regions dominated by wear particles or fibrous tissue rather than inflamed areas, histology may reveal a combined (type III) or indifferent (type IV) membrane despite active infection [31]. In addition, interpretation variability must be acknowledged. While the Krenn and Morawietz classification provides a standardized framework, overlap exists between categories, and interobserver differences have been reported [50].

Our findings align with previous studies such as Renz et al. (2023), who analysed acute hematogenous PJIs using the same classification and also observed type II and III membranes as indicative of infection [51]. However, that study did not differentiate the membrane distribution specifically for DAIR-treated cases, and overall, the literature on the use of this classification in the specific context of DAIR remains sparse to non-existent. In our cohort, we found no significant difference in the distribution of membrane types between hips and knees treated with DAIR, but we did observe a strong correlation between Tsukayama type II– IV infections and Krenn and Morawietz type II and III membranes (infectious and mixed), confirming the system’s diagnostic value in the acute setting.

However, our data suggest that the Krenn and Morawietz classification cannot deliver prognostic value for PJI recurrence or DAIR outcome. The high proportion of type IV (indifferent) and type III (combined) membranes does not contradict the confirmed diagnosis but rather illustrates the diagnostic limitations of histology alone.

### Limitations

This study has several limitations. First, it is a retrospective analysis with inherent constraints such as potential selection and documentation bias. In addition, not all patients could be followed up completely, resulting in a relatively high lost-to-follow-up rate of 23.6%. Finally, this is a single-center study, at a tertiary endoprosthetic referral center, where patients are often transferred to in case of complications (e.g., due to patient-related factors such as multimorbidity). Therefore, the results may not be directly generalizable to other healthcare settings.

## Conclusion

In this single-center retrospective study at an endoprothetic referral center, we found that the success of DAIR for acute periprosthetic joint infection remains limited, with a revision-free implant survival rate of only 42.6% for hips and 29.7% for knees at 24 months. Advanced patient age, a higher ASA score, and the presence of revision implants were associated with poorer outcomes, confirming known risk factors for treatment failure. The type of infection according to the Tsukayama classification did not influence implant survival. Despite following a standardized diagnostic and therapeutic algorithm, preoperative synovial cell count and CRP levels did not reliably predict DAIR success. Our findings highlight that patient-related risk factors—many of which are non-modifiable—continue to play a major role in treatment outcomes. These results emphasize the importance informed decision-making when considering DAIR, particularly in patients with revision implants or significant comorbidities.

## Data Availability

The original contributions presented in this study are included in the article; further inquiries can be directed to the corresponding author.
